# Breakfast skipping and steroid withdrawal in ulcerative colitis: A population‐based study in Japan

**DOI:** 10.14814/phy2.70708

**Published:** 2025-12-26

**Authors:** Hiroshi Fukuda, Haru Miyagi, Kenta Yamamoto

**Affiliations:** ^1^ Faculty of Pharmaceutical Sciences Teikyo Heisei University Tokyo Japan

**Keywords:** breakfast skipping, health checkup, intermittent fasting, steroid withdrawal, ulcerative colitis

## Abstract

Ulcerative colitis (UC) is a chronic inflammatory bowel disease with increasing prevalence in Japan. While pharmacological therapies have advanced, the effect of dietary timing, particularly breakfast skipping, on disease course remains unclear. We conducted a retrospective cohort study using the JMDC database (2015–2023), which contains claims and annual health checkup data from about 8.17 million insured individuals. We identified 1645 UC patients who initiated corticosteroid therapy within 1 year after diagnosis and were followed for at least 3 years. Breakfast skipping was defined as skipping breakfast more than three times per week. The primary outcome was steroid withdrawal, defined as discontinuation of corticosteroid prescriptions. Logistic regression models estimated odds ratios (ORs) and 95% confidence intervals (CIs) for steroid withdrawal according to breakfast habits. Among 1645 patients, 303 (18.4%) reported skipping breakfast. Steroid withdrawal occurred in 543 patients (33.0%), including 108 (35.6%) in the breakfast skipping group and 435 (32.4%) in the breakfast consuming group. In fully adjusted models accounting for 5‐aminosalicylic acid and immunosuppressant use, breakfast skipping was associated with higher odds of steroid withdrawal (OR 1.52, 95% CI 1.08–2.14). These findings suggest that meal timing may influence sustained remission in UC.

## 1 INTRODUCTION

Ulcerative colitis (UC) is a chronic inflammatory bowel disease characterized by relapsing and remitting courses, with increasing prevalence in Japan. Annual prevalence of UC has risen from five per 100,000 population in 2010 to 98 per 100,000 in 2019, reflecting a substantial public health burden (Yamazaki et al., 2023). Although pharmacological treatments, including 5‐aminosalicylic acid (5‐ASA), corticosteroids, and immunosuppressants, have improved disease management, non‐pharmacological factors, particularly dietary habits, remain insufficiently understood.

Dietary management for UC has not been established as a standardized guideline. Nevertheless, certain dietary patterns, such as high‐fat or highly processed food intake, have been implicated in UC risk and exacerbations (Dong et al., 2022; Li et al., 2020; Narula et al., 2021). In recent years, intermittent fasting has attracted attention for its potential benefits in reducing systemic inflammation and improving gut homeostasis (Paukkonen et al., 2024).

Breakfast skipping is generally regarded as a harmful lifestyle behavior due to its association with metabolic disorders, sarcopenia, and circadian rhythm disruption (Kiriyama et al., 2022). However, emerging evidence suggests that short‐term fasting may also exert favorable effects on intestinal microbiota, autophagy activation, and inflammatory control (Rangan et al., 2019). The role of breakfast skipping in UC, however, has not been systematically evaluated.

In this study, we investigated the association between breakfast skipping and sustained remission in patients with UC using a large‐scale health checkup and claims database in Japan. Specifically, we examined whether patients who reported skipping breakfast more than three times per week were more likely to achieve steroid withdrawal compared with those who regularly consumed breakfast.

## 2 MATERIALS AND METHODS

This study was approved by the Ethics Committee of Teikyo Heisei University (approval number: 2021‐054‐1). The need for informed consent was waived because the database is anonymized.

### 2.1 Study population

We used the JMDC database (January 2015–June 2023), which includes claims and annual health checkup data from approximately 8.17 million individuals insured under employment‐based health insurance in Japan (Nagai et al., 2021). A total of 65,115 individuals diagnosed with UC (ICD‐10 code K51) were identified.

We included patients who received corticosteroid therapy during the first year after diagnosis and had continuous treatment records for at least 3 years. After excluding those without a 6‐month screening period before the first diagnosis, 3566 patients remained. Among them, 2802 patients underwent a health checkup within 1 year prior to diagnosis. After excluding those with missing baseline data on demographic, anthropometric, laboratory, and lifestyle factors, 2057 patients were eligible. Finally, we restricted the analysis to patients who either (a) discontinued corticosteroid therapy after the second year and maintained nonsteroid treatment in the third year, or (b) continued corticosteroid therapy throughout the 3 years, in order to exclude patients with temporary discontinuation and reinitiation of steroid therapy (Table 4). This yielded a final analytic cohort of 1645 patients.

Sex (male/female) was obtained from health checkup records and included as a covariate in all analyses. Gender identity was not assessed in this database.

### 2.2 Outcomes

The primary outcome of this study was steroid withdrawal. Steroid withdrawal was defined as discontinuation of corticosteroid prescriptions during the follow‐up period. The outcome was compared between individuals with skipping breakfast and with regularly consumed breakfast. Breakfast skipping was defined by a positive response to the item: “Do you skip breakfast more than three times per week?” using a standardized questionnaire administered as part of the Specific Health Checkups, an annual health screening and promotion service mandated by the Japanese Ministry of Health, Labour and Welfare (Kaneko et al., 2020). These checkups follow a nearly uniform protocol for most employees in Japan.

### 2.3 Covariates

Potential covariates were selected from factors thought to influence steroid withdrawal. These consisted of age, sex, waist circumference, smoking, late‐night dinner, and medication use (5‐ASA, immunosuppressants) during the first year of treatment. Current smoking and late‐night dinner were assessed from a self‐administered questionnaire during the Specific Health Checkups. The questionnaire items are summarized in Table 1.

**TABLE 1:** Self‐administered questionnaire during the specific health checkups.

Lifestyle behaviors	Questionnaire items	Answer options
Breakfast skipping	Do you skip breakfast more than three times per week?	Yes or No
Late‐night dinner	Do you eat dinner within 2 h of going to bed on three or more days per week?	Yes or No
Current smoker	Are you currently a regular smoker?	Yes or No

### 2.4 Statistical analysis

Baseline characteristics were summarized as mean (standard deviation) for continuous variables, median (interquartile range) for triglycerides, and frequency (%) for categorical variables. Intergroup differences at baseline were analyzed using the unpaired t‐test for continuous variables (except triglyceride) and the χ^2^ test for categorical variables. Triglyceride was analyzed using the Wilcoxon test. Logistic regression models were used to estimate odds ratios (ORs) and 95% confidence intervals (CIs) for steroid withdrawal according to breakfast skipping status.

Model 1: adjusted for age, sex, waist circumference, smoking, and late‐night dinner.

Model 2: further adjusted for 5‐ASA and immunosuppressant use during the first year.

Statistical significance was defined as a *p* value of less than 0.05. All statistical analyses were conducted using JMP Pro version 17 (SAS Institute Inc., Cary, NC, USA).

## 3 RESULTS

Table 2 presents the baseline characteristics of the participants. Of the 1645 eligible patients, 303 (18.4%) reported skipping breakfast ≥3 times per week. Compared with breakfast consumers, breakfast skippers were younger, more likely to be male, and had higher proportions of smoking and late‐night dinner habits (Table 2).

**TABLE 2:** Baseline characteristics of patients according to breakfast skipping status.

	Breakfast skipping		*p* Value
	Yes	No	
*N*	303	1342	
Age (years)	41 (9.9)	45 (9.5)	<0.0001
Sex (male)	229 (75.6)	902 (67.2)	0.0045
Body mass index (kg/m^2^)	23.3 (4.09)	22.9 (3.66)	0.0858
Waist circumference (cm)	82.6 (10.97)	81.5 (9.78)	0.0721
Systolic blood pressure (mmHg)	118 (13.6)	118 (15.1)	0.9712
Diastolic blood pressure (mmHg)	73 (10.4)	73 (11.2)	0.7514
LDL‐cholesterol (mg/dL)	121 (29.4)	118 (29.7)	0.1198
HDL‐cholesterol (mg/dL)	59 (15.6)	62 (16.7)	0.0062
Triglycerides (mg/dL)	86 (58–133)	84 (60–120)	0.6086
HbA1c (%)	5.4 (0.48)	5.5 (0.42)	0.0304
Fasting blood glucose (mg/dL)	94 (13.6)	93 (12.5)	0.2485
Current smoker	100 (33.0)	277 (20.6)	<0.0001
Late‐night dinner	172 (56.8)	442 (32.9)	0.0001
Treatment during the first year			
Corticosteroids	303 (100.0)	1342 (100.0)	—
5‐ASA	206 (53.8)	856 (51.1)	0.3490
immunosuppressants	50 (13.1)	165 (9.9)	0.0650

*Note*: Triglycerides are presented as medians (interquartile ranges). Other data are presented as means (standard deviations) or as numbers (%).

Abbreviations: 5‐ASA, 5‐aminosalicylic acid; HbA1c, hemoglobin A1c; HDL, high‐density lipoprotein; LDL, low‐density lipoprotein.

Table 3 presents the odds ratios of steroid withdrawal according to breakfast skipping status. Overall, 543 patients (33.0%) achieved steroid withdrawal within 3 years. The proportion of steroid withdrawal was 35.6% among breakfast skippers and 32.4% among breakfast consumers. In crude analysis, breakfast skipping was not significantly associated with steroid withdrawal. After adjustment for age, sex, waist circumference, smoking, and late‐night dinner (Model 1), the association remained nonsignificant. However, after further adjustment for 5‐ASA and immunosuppressant use (Model 2), breakfast skipping was significantly associated with steroid withdrawal.

**TABLE 3:** Odds ratios for steroid withdrawal according to breakfast skipping status.

Breakfast skipping	*N*	Steroid withdrawal	Crude OR (95% CI)	Model 1 OR (95% CI)	Model 2 OR (95% CI)
		*n* (%)			
No	1342	435 (32.4)	1.00 (Reference)	1.00 (Reference)	1.00 (Reference)
Yes	303	108 (35.6)	1.15 (0.89–1.50)	1.22 (0.93–1.61)	1.52 (1.08–2.14)
*p* Value		0.2803	0.2805	0.1468	0.0159

*Note*: Model 1: adjusted for age, sex, waist circumference, smoking, and late‐night dinner. Model 2: further adjusted for 5‐ASA and immunosuppressant use during the first year.

Abbreviation: 5‐ASA, 5‐aminosalicylic acid; CI, confidence interval; OR, odds ratio.

Table 4 shows the prescribed medications during the 3‐year follow‐up. A higher proportion of patients in the corticosteroid continuation group received 5‐ASA and immunosuppressants compared with the steroid withdrawal group.

**TABLE 4:** Classes of medications prescribed during the 3‐year follow‐up.

	Steroid continuation	Steroid withdrawal
Corticosteroids		
Year 1	1102 (100)	543 (100)
Year 2	1102 (100)	0 (0.0)
Year 3	1102 (100)	0 (0.0)
5‐ASA		
Year 1	857 (77.8)	103 (19.0)
Year 2	857 (77.8)	0 (0.0)
Year 3	857 (77.8)	22 (4.1)
Immunosuppressants		
Year 1	201 (18.2)	3 (0.6)
Year 2	201 (18.2)	0 (0.0)
Year 3	201 (18.2)	2 (0.4)

*Note*: Data are presented as numbers (%).

Abbreviation: 5‐ASA, 5‐aminosalicylic acid.

Table 5 summarizes the classes of medications prescribed and the number (percentage) of patients receiving each. Among corticosteroids, prednisolone was the most commonly prescribed, followed by betamethasone and dexamethasone. For 5‐ASA, mesalazine was the predominant agent, while azathioprine was the most frequently prescribed immunosuppressant.

**TABLE 5:** Prescribed corticosteroids, 5‐ASA, and immunosuppressants classification.

	Year 1	Year 2	Year 3
Corticosteroids			
Prednisolone	726 (44.1)	316 (19.2)	248 (15.1)
Betamethasone	528 (32.1)	204 (12.4)	165 (10.0)
Dexamethasone	391 (23.8)	154 (9.4)	146 (8.9)
Combination of systemic corticosteroids	273 (16.6)	111 (6.7)	101 (6.1)
Hydrocortisone	85 (5.2)	43 (2.6)	42 (2.6)
Methylprednisolone	62 (3.8)	24 (1.5)	23 (1.4)
5‐ASA			
Mesalazine	932 (56.7)	768 (46.7)	779 (47.4)
Sulfasalazine	76 (4.6)	38 (2.3)	35 (2.1)
Immunosuppressants/Biologics			
Azathioprine	142 (8.6)	165 (10.0)	165 (10.0)
Infliximab	51 (3.1)	63 (3.8)	56 (3.4)
Adalimumab	33 (2.0)	39 (2.4)	39 (2.4)
Vedolizumab	21 (1.3)	28 (1.7)	42 (2.6)
Golimumab	13 (0.8)	18 (1.1)	16 (1.0)
Tofacitinib	10 (0.6)	24 (1.5)	25 (1.5)
Ustekinumab	7 (0.4)	19 (1.2)	30 (1.8)
Upadacitinib	0 (0.0)	2 (0.1)	4 (0.2)
Filgotinib	0 (0.0)	0 (0.0)	4 (0.2)

*Note*: Data are presented as numbers (%).

Abbreviation: 5‐ASA, 5‐aminosalicylic acid.

## 4 DISCUSSION

In this epidemiological study, we found that breakfast skipping was associated with higher odds of steroid withdrawal in patients with UC. After adjustment for the covariates (Model 2), the odds of steroid withdrawal were approximately 50% higher among breakfast skippers compared with breakfast consumers. These findings suggest that breakfast skipping may contribute to sustaining remission in UC. To our knowledge, this is the first epidemiological study to evaluate breakfast skipping in relation to steroid withdrawal and sustained remission in patients with UC.

The association between breakfast skipping and steroid withdrawal appeared only after adjustment for 5‐ASA and immunosuppressant use in Model 2, whereas the crude and partially adjusted models did not show statistical significance. Disease severity is a strong determinant of successful steroid withdrawal, and although we attempted to account for its influence as much as possible by using 5‐ASA and immunosuppressant use as proxy indicators, the database does not allow for a precise assessment of clinical severity. Consequently, the observed association between breakfast skipping and steroid withdrawal may reflect not only the direct effect of breakfast habits but also residual confounding related to disease severity. Moreover, while medication use serves as a proxy for severity, it may also be indirectly associated with underlying lifestyle factors, raising the possibility that including these variables as covariates could unintentionally alter the underlying group structure. Therefore, the changes in the effect estimates after adjusting for these treatment‐related variables should be interpreted with caution, and future studies should evaluate the consistency of these findings using models that incorporate more accurate measures of disease severity.

Our results add to the growing body of literature suggesting that dietary timing and fasting regimens influence chronic inflammatory diseases. Experimental studies have demonstrated that intermittent fasting can modulate gut microbiota, enhance intestinal regeneration, and reduce pro‐inflammatory immune cell activity (Luo et al., 2024; Paukkonen et al., 2024; Rangan et al., 2019). Consistent with these mechanisms, our findings imply that extending the overnight fasting window by skipping breakfast may alleviate chronic intestinal inflammation and support disease remission.

The dietary composition of individuals who consume breakfast varies widely, and its specific content cannot be assessed from health checkup data. Previous studies have shown that Western‐style dietary patterns, including bread, butter, margarine, cheese, red meat, and processed meat products, are associated with an increased risk of UC (Li et al., 2020). Higher intake of meat and red meat (Dong et al., 2022) and frequent consumption of ultra‐processed foods (Narula et al., 2021) have also been linked to a greater risk of UC. These findings suggest that if breakfast includes foods known to adversely affect UC, consuming such meals could potentially worsen the disease. Therefore, it may be necessary to consider that, for individuals whose typical breakfast consists of foods associated with a higher risk of exacerbating UC, skipping breakfast might be more favorable than consuming such meals.

Breakfast skipping has been widely linked to adverse health outcomes in the general population, including metabolic syndrome, obesity, and sarcopenia (Kiriyama et al., 2022; Monzani et al., 2019). Thus, breakfast skipping cannot be universally recommended as a beneficial lifestyle habit. Moreover, breakfast skippers in this study exhibited additional unhealthy behaviors, including higher rates of smoking and late‐night eating, which complicate the attribution of the observed association solely to breakfast habits. Experimental studies have shown that structured dietary restriction such as fasting‐mimicking diets (FMDs) may have beneficial biological effects (Rangan et al., 2019). FMD cycles have been reported to reduce cancer incidence and aging‐related immunosuppression through hematopoietic stem‐cell–mediated regeneration (Brandhorst et al., 2015; Cheng et al., 2014) and to ameliorate or even reverse disease progression in mouse models of multiple sclerosis and type 1 and type 2 diabetes (Cheng et al., 2017; Choi et al., 2016). Given these findings, it would be of interest to examine how controlled fasting regimens such as FMD relate to steroid withdrawal and the maintenance of remission in patients with UC.

Several limitations should be considered when interpreting our findings. First, breakfast skipping was assessed using a single dichotomous self‐reported item without information on dietary content, caloric intake, or the reasons for skipping breakfast. Individuals may skip breakfast for various reasons, including UC‐related symptoms, lifestyle habits, or work schedules, which could differentially influence disease activity. Therefore, we could not determine whether the observed association was affected by the specific types of breakfast consumed or by the underlying motivations for skipping breakfast. Second, because this was an observational study, residual confounding cannot be excluded, and causality cannot be established. Disease severity could not be directly assessed using the available claims and checkup data, and the use of medications such as 5‐ASA and immunosuppressants only partially reflects underlying severity. In addition, decisions regarding steroid tapering and withdrawal may vary among clinicians and institutions, introducing further heterogeneity that could not be fully controlled. Finally, the study population mainly comprised employed and insured individuals in Japan, potentially limiting the generalizability of our findings to other populations or healthcare settings. Providing more comprehensive dietary assessments and objective measures of disease severity in future studies will help to clarify the mechanisms underlying the observed associations.

In conclusion, breakfast skipping was associated with sustained remission as indicated by steroid withdrawal in patients with UC. These findings suggest that dietary timing may play a role in disease management. Further prospective and interventional studies are warranted to clarify the mechanisms and to evaluate the safety and applicability of fasting‐based dietary strategies in UC.

## AUTHOR CONTRIBUTIONS

H.F. and K.Y. formulated the research questions and designed the study; H.F. and H.M. analyzed the data; H.F., H.M., and K.Y. performed data interpretation; H.F. and K.Y. drafted the manuscript; K.Y. had the primary responsibility for the final contents; and all authors read and approved the final manuscript.

## FUNDING INFORMATION

This study was supported by the JSPS KAKENHI grant (grant numbers: JP20K11422, JP2011422S, and JP23K10722) to K. Yamamoto.

## CONFLICT OF INTEREST STATEMENT

The authors declare no competing interests.

## ETHICS STATEMENT

All methods were performed in accordance with the relevant guidelines and regulations, and the study was conducted in compliance with the Declaration of Helsinki.

## Data Availability

The data that support the findings of this study are available from JMDC, Inc.; however, restrictions apply to the availability of these data, which were used under license for the current research and therefore are not publicly available.
